# Association of Vitamin B12 and Ferritin Levels With Neuropathic Pain and Disease Severity in Fibromyalgia Syndrome

**DOI:** 10.7759/cureus.105258

**Published:** 2026-03-15

**Authors:** Habiba Pervaz, Ahmad Rafique, Hafiz Hamyoun I Khan, Rafia Javed, Saba Arif, Syed Sufyan Khaliq, Saqlain Ghazanfar, Abdullah Ishfaq

**Affiliations:** 1 Internal Medicine, Southport and Formby District General Hospital, Southport, GBR; 2 Internal Medicine, Farooq Hospital - West Wood Branch, Lahore, PAK; 3 General Practice, Shaikh Zayed Hospital, Lahore, PAK; 4 Biochemistry, Allama Iqbal Medical College, Lahore, PAK; 5 Biochemistry, Akhtar Saeed Medical and Dental College Lahore, Lahore, PAK; 6 Emergency, Lady Reading Hospital Medical Teaching Institute, Peshawar, PAK; 7 Internal Medicine, Rawalpindi Medical University, Rawalpindi, PAK; 8 Accident and Emergency, Shaikh Zayed Hospital, Lahore, PAK

**Keywords:** biomarkers, chronic pain, ferritin, fibromyalgia syndrome, neuropathic pain, pain severity, vitamin b12

## Abstract

Background: Fibromyalgia syndrome (FMS) is a chronic disorder characterized by widespread musculoskeletal pain, often accompanied by fatigue, sleep disturbance, and cognitive impairment. Neuropathic pain is an important but underrecognized component of FMS, which significantly worsens disease burden. Micronutrient deficiencies, particularly serum vitamin B12 and ferritin, are suggested to influence pain perception and neuronal function, yet their role in FMS remains unclear, especially in Pakistan.

Objectives: To determine the association of serum vitamin B12 and ferritin levels with neuropathic pain and disease severity in patients with fibromyalgia.

Study design and setting: A cross-sectional analytical study conducted at the Department of Medicine, Shaikh Zayed Hospital, Lahore, Pakistan, from January to June 2025.

Methodology: A total of 241 patients fulfilling the American College of Rheumatology (ACR 2016) criteria for FMS were enrolled. Demographic data, Fibromyalgia Impact Questionnaire-Revised (FIQR) scores, and Douleur Neuropathique en 4 Questions (DN4) scores were recorded. Serum vitamin B12 and ferritin levels were measured. Independent sample t-test and chi-square test were applied for comparisons, and multivariable logistic regression was performed to identify predictors of neuropathic pain, with p < 0.05 considered significant.

Results: Mean age of participants was 46.0 ± 15.6 years, with female predominance (77.6%). Neuropathic pain was present in 58.5% of cases. Patients with neuropathic pain had significantly higher FIQR (52.7 ± 9.5 vs. 41.0 ± 8.9, p < 0.001) and DN4 scores (5.8 ± 1.4 vs. 2.8 ± 1.0, p < 0.001). Serum vitamin B12 and ferritin levels were lower in neuropathic patients (p = 0.001 and p = 0.005, respectively). Multivariable regression identified FIQR score as the strongest predictor of neuropathic pain (OR = 1.159, 95% CI: 1.112-1.208, p < 0.001).

Conclusion: Neuropathic pain is highly prevalent in FMS and is strongly associated with disease severity. Although vitamin B12 and ferritin showed lower levels in affected patients, only disease severity independently predicted neuropathic pain. Larger longitudinal studies are warranted to clarify causal pathways and therapeutic implications.

## Introduction

Fibromyalgia syndrome (also called FMS) is a persistent, complex pain illness distinguished by pervasive musculoskeletal discomfort, sleep disturbance, persistent fatigue, cognitive dysfunction, and various somatic symptoms [1]. The condition is increasingly understood as a dysfunction of pain processing rather than a primary peripheral musculoskeletal disease, with central sensitization and altered nociceptive modulation playing major roles [2,3]. Neuropathic manifestations - including paresthesia, dysesthesia, and features of small-fiber neuropathy - are common and contribute to higher symptom severity and reduced quality of life [4]. Globally, FMS affects approximately 1-5% of adults, with variation depending on diagnostic criteria and population under study [5]. A hospital-based study conducted in Pakistan reported a prevalence of fibromyalgia of approximately 33.3% among patients presenting to a tertiary care hospital setting. However, this estimate reflects findings from a clinical population and should not be interpreted as representative of the prevalence in the general population [6].

The etiopathogenesis of FMS is complex and multifactorial, involving genetic predisposition, psychosocial stress, sleep disturbance, persistent nociceptive input, and neuroimmune interactions [7]. Micronutrient insufficiencies - particularly vitamin B12 and iron (reflected by serum ferritin) - have recently gained attention due to their roles in neuronal function, myelin synthesis, neurotransmitter regulation, and central pain modulation [8,9]. Pathophysiological models indicate elevated excitatory neurotransmitters, impaired descending inhibition, and neuroinflammation, while small-fiber neuropathy may coexist in a subset of patients - mechanisms potentially worsened by micronutrient dysregulation [10,11]. Diagnosis is primarily clinical, supported by validated tools such as the American College of Rheumatology (ACR) and Analgesic, Anesthetic, and Addiction Clinical Trial Translations, Innovations, Opportunities, and Networks (AAPT) criteria, with laboratory investigations targeted to exclude alternative diagnoses and evaluate contributory deficiencies such as low vitamin B12 and ferritin in cases with neuropathic symptoms or fatigue. Management is multidisciplinary, combining education, exercise, psychological therapy, and pharmacologic measures for symptom control [12]. Epidemiological studies indicate that neuropathic pain characteristics may be present in nearly 30-50% of individuals with fibromyalgia, highlighting the importance of evaluating neuropathic components in this population. These findings support the hypothesis that underlying neurological alterations may contribute to symptom severity and pain perception in fibromyalgia, thereby providing a rationale for investigating biological factors, including micronutrient status, that may influence neuropathic pain manifestations [12].

In the present study, neuropathic pain was evaluated as a clinical feature within the fibromyalgia population rather than as a separate reference condition. This approach was adopted to better understand the relationship between neuropathic pain characteristics and potential biological factors, particularly vitamin B12 and ferritin levels, that may influence symptom severity in patients with FMS. Given the biological plausibility linking micronutrient imbalance with neuronal dysfunction and the high frequency of neuropathic symptoms in FMS, it is hypothesized that deficiencies in vitamin B12 and ferritin may be associated with increased neuropathic pain and greater disease severity. Therefore, investigating the association between vitamin B12 and ferritin levels, neuropathic pain, and disease severity may help identify potentially modifiable contributors to patient outcomes. This study addresses a major knowledge gap in Pakistan and seeks to provide evidence that may support practical and low-cost strategies for improving the management of patients with fibromyalgia.

## Materials and methods

This cross-sectional analytic study was performed in the Department of Medicine, Shaikh Zayed Hospital, Lahore, Pakistan, from January to June 2025, following ethical approval from the Institutional Review Board (SZMC/TERC/381/24, dated 23/07/2024). Those individuals who met the ACR 2016 criteria for FMS were included in the recruitment process.

Sample size calculation

The sample size was calculated using the formula for estimating a single population proportion. A prevalence of 33.3% reported in a hospital-based study was used for the calculation, with a 90% confidence level (Z = 1.645) and a margin of error of 5% (d = 0.05). Based on these parameters, the required sample size was calculated to be 241 participants. A non-probability consecutive sampling technique was used, whereby all eligible patients meeting the inclusion criteria during the study period were recruited until the required sample size was achieved.

Inclusion and exclusion criteria

All adult patients aged between 18 and 60 years, fulfilling the ACR 2016 diagnostic criteria for FMS, were eligible for inclusion in this study. Participants must present with clinical features consistent with fibromyalgia, including widespread musculoskeletal pain of at least three months’ duration, and should provide informed consent to participate.

Patients were excluded if they had known systemic or metabolic disorders that can independently influence neuropathic pain or micronutrient status, such as diabetes mellitus, hypothyroidism, chronic kidney disease, chronic liver disease, or malignancy. Those with documented neurological disorders, including peripheral neuropathies of other causes, multiple sclerosis, or Parkinson’s disease, were also excluded. Additional exclusion criteria include pregnancy or lactation, current use of vitamin B12 or iron supplementation within the past three months, history of major psychiatric illness that could interfere with symptom reporting, or inability to comply with study procedures.

Data collection procedure

At the time of recruitment, a structured interview was conducted to obtain demographic information, including age, gender, educational status, occupation, and socioeconomic background. Detailed clinical history regarding the duration of musculoskeletal pain, fatigue, sleep disturbance, cognitive complaints, and associated comorbidities was recorded. A thorough physical and rheumatologic examination was performed to confirm the diagnosis according to the 2016 criteria set by the ACR for fibromyalgia.

Following clinical assessment, disease severity was evaluated using the Fibromyalgia Impact Questionnaire-Revised (FIQR). This validated instrument consisted of 21 items covering three domains: function, overall impact, and symptoms. Scores were computed and expressed on a 0-100 scale, with higher values indicating greater severity [13]. Neuropathic pain features were screened using the Douleur Neuropathique en 4 Questions (DN4) questionnaire, a validated screening tool composed of 10 items relating to pain descriptors and sensory examination. A score ≥4 was considered indicative of the presence of neuropathic pain characteristics [14]. Both scales were administered by trained investigators to ensure consistency.

For biochemical analysis, 5 mL of venous blood was taken in an aseptic manner from each participant. Samples were transported promptly to the hospital diagnostic laboratory. Serum was separated by centrifugation and stored at a controlled temperature until analysis. Serum vitamin B12 and serum ferritin levels were measured using automated chemiluminescent immunoassay analysers in accordance with the directions provided by the maker. Methods for both internal and external quality assurance were utilized to maintain the reliability of laboratory results. All collected data, including demographic variables, clinical characteristics, FIQR and DN4 scores, and laboratory findings, were entered into a predesigned proforma. Data collection was supervised by the principal investigator to ensure completeness and accuracy at every stage.

Statistical analysis

The data was examined with the help of IBM SPSS Statistics for Windows, Version 25 (Released 2017; IBM Corp., Armonk, New York, United States). Numerical variables included age, serum vitamin B12 levels, ferritin levels, FIQR scores, and DN4 scores, and were presented as mean ± standard deviation. Categorical variables included gender, presence of neuropathic pain, and disease severity categories, and were expressed as frequencies and percentages. The normality of continuous variables was assessed using the Shapiro-Wilk test. If the data were found to be normally distributed, independent sample t-tests were applied for group comparisons; however, if the data were not normally distributed, the Mann-Whitney U test was used as the non-parametric alternative. For continuous variables, we used an independent sample t-test; for categorical variables, we used a chi-square test. A p-value < 0.05 was taken as statistically significant. Data were stratified with respect to age, gender, and disease severity to address potential effect modifiers. After stratification, categorical variables were tested using the chi-square test and continuous variables with the independent sample t-test, with a p-value less than 0.05 being considered statistically significant.

Operational definitions

Fibromyalgia Syndrome (FMS)

FMS was diagnosed according to the ACR 2016 diagnostic criteria, which incorporate the Widespread Pain Index (WPI) and Symptom Severity Scale (SSS) [15]. Patients were classified as having FMS if their SSS score was 5 or higher and their WPI score was 7 or higher, or if their WPI score was 3-6 and their SSS score was 9 or higher, and if their symptoms persisted for three months or longer and could not be explained by any other medical illness.

Neuropathic Pain

Neuropathic pain features were screened using the DN4 questionnaire, a validated screening tool composed of 10 items relating to pain descriptors and sensory examination. A score ≥4 was considered indicative of the presence of neuropathic pain characteristics.

Disease Severity

FIQR was used to evaluate the severity of the disease. Scores were classified as mild (≤39), moderate (40-59), and severe (≥60).

Vitamin B12 Deficiency

Serum vitamin B12 levels were measured, and the patients were classified as either deficient (<200 pg/mL), borderline (200-300 pg/mL), or normal (>300 pg/mL) based on standard laboratory cut-offs.

Ferritin Deficiency

Serum ferritin levels were evaluated and considered deficient (<30 ng/mL) or normal (≥30 ng/mL) according to accepted reference ranges.

## Results

The study included 241 participants with a mean age of 46.0 ± 15.6 years, of whom the majority were above 40 years (143, 59.3%). Females constituted a larger proportion of the study population (187, 77.6%) compared to males (54, 22.4%). Neuropathic pain was present in 141 (58.5%) participants. The mean FIQR score was 47.8 ± 10.9, and the mean DN4 score was 4.6 ± 1.9. Regarding biochemical parameters, the mean serum vitamin B12 level was 288.5 ± 69.3 pg/mL, with 21 (8.7%) participants classified as deficient. Similarly, the mean ferritin level was 63.2 ± 27.2 ng/mL, with 27 (11.2%) having deficiency. These findings highlight the demographic, clinical, and biochemical distribution of the study population, as given in Table 1.

**Table 1:** Demographic and baseline clinical characteristics of study participants (n = 241) Data are presented as mean ± standard deviation (SD) for continuous variables and as number (percentage) (n (%)) for categorical variables. FIQR: Fibromyalgia Impact Questionnaire-Revised; DN4: Douleur Neuropathique en 4 Questions

Variable	Category	Mean ± SD/n (%)
Age (years)	Overall	46.0 ± 15.6
	18-40 years	98 (40.7)
	>40 years	143 (59.3)
Gender	Male	54 (22.4)
	Female	187 (77.6)
Neuropathic pain	Yes	141 (58.5)
	No	100 (41.5)
FIQR score	Overall	47.8 ± 10.9
DN4 score	Overall	4.6 ± 1.9
Vitamin B12 (pg/mL)	Overall	288.5 ± 69.3
	Deficient (<200 pg/mL)	21 (8.7)
	Normal	220 (91.3)
Ferritin (ng/mL)	Overall	63.2 ± 27.2
	Deficient (<30 ng/mL)	27 (11.2)
	Normal	214 (88.8)

Mean DN4 scores were higher among participants classified as having neuropathic pain characteristics according to the DN4 threshold. Comparison of continuous variables between participants with and without neuropathic pain revealed no significant difference in mean age between groups (p = 0.461). However, patients with neuropathic pain had significantly higher FIQR scores (52.7 ± 9.5 vs. 41.0 ± 8.9, p < 0.001) and DN4 scores (5.8 ± 1.4 vs. 2.8 ± 1.0, p < 0.001). In terms of biochemical markers, mean serum vitamin B12 levels were lower in patients with neuropathic pain compared to those without (275.8 ± 66.8 vs. 308.1 ± 69.9 pg/mL, p = 0.001). Similarly, mean serum ferritin levels were also significantly lower in patients with neuropathic pain (59.1 ± 26.1 vs. 68.9 ± 27.2 ng/mL, p = 0.005). These results demonstrate a strong association of disease severity and biochemical deficiencies with neuropathic pain, as presented in Table 2.

**Table 2:** Comparison of continuous variables by presence of neuropathic pain An independent sample t-test was used for comparison of continuous variables. P-value < 0.05 was considered statistically significant. Significant p-values are marked with an asterisk (*). FIQR: Fibromyalgia Impact Questionnaire-Revised; DN4: Douleur Neuropathique en 4 Questions

Variable	Neuropathic Yes (Mean ± SD)	Neuropathic No (Mean ± SD)	t-value	p-value
Age (years)	45.4 ± 15.3	46.9 ± 16.0	0.74	0.461
FIQR score	52.7 ± 9.5	41.0 ± 8.9	10.82	<0.001*
DN4 score	5.8 ± 1.4	2.8 ± 1.0	18.91	<0.001*
Vitamin B12 (pg/mL)	275.8 ± 66.8	308.1 ± 69.9	3.41	0.001*
Ferritin (ng/mL)	59.1 ± 26.1	68.9 ± 27.2	2.82	0.005*

The association between categorical variables and neuropathic pain revealed significant gender differences, with neuropathic pain more common among females (124/187, 66.3%) than males (17/54, 31.5%) (p < 0.001). Vitamin B12 deficiency showed neuropathic pain in nine (42.9%) deficient participants compared to 132 (60.0%) non-deficient individuals, but this difference did not reach statistical significance (p = 0.357). Similarly, ferritin deficiency was associated with neuropathic pain in nine (33.3%) participants compared to 132 (61.7%) participants with normal ferritin levels, though this finding was also not statistically significant (p = 0.076). Disease severity based on FIQR demonstrated a strong and graded association with neuropathic pain, where severe cases showed neuropathic pain in 26 (89.7%) participants compared to 85 (58.2%) moderate and 30 (45.5%) mild cases, with statistical significance (p < 0.001). These findings suggest that female gender and greater disease severity are strongly linked with the presence of neuropathic pain, as shown in Table 3.

**Table 3:** Association of categorical variables with neuropathic pain Chi-square test was used to assess the association between categorical variables and neuropathic pain. P-value < 0.05 was considered statistically significant. Significant values are marked with an asterisk (*). FIQR: Fibromyalgia Impact Questionnaire-Revised

Variable	Category	Neuropathic Yes, n (%)	Neuropathic No, n (%)	χ^2^ value*	p-value
Gender	Male	17 (31.5)	37 (68.5)	χ^2^ = 18.62	<0.001*
	Female	124 (66.3)	63 (33.7)		
Vitamin B12 status	Deficient (<200 pg/mL)	9 (42.9)	12 (57.1)	χ^2^ = 0.85	0.357
	Normal	132 (60.0)	88 (40.0)		
Ferritin status	Deficient (<30 ng/mL)	9 (33.3)	18 (66.7)	χ^2^ = 3.15	0.076
	Normal	132 (61.7)	82 (38.3)		
FIQR severity	Mild	30 (45.5)	36 (54.5)	χ^2^ = 29.48	<0.001*
	Moderate	85 (58.2)	61 (41.8)		
	Severe	26 (89.7)	3 (10.3)		

Multivariable logistic regression analysis was performed to identify predictors of neuropathic pain. Increasing age was not significantly associated with neuropathic pain (OR = 0.993, 95% CI: 0.974-1.013, p = 0.502). Gender also did not demonstrate a significant association (OR = 1.102, 95% CI: 0.524-2.318, p = 0.798). Among all predictors, FIQR score emerged as a significant factor, where each unit increase in FIQR was associated with a 15.9% higher likelihood of neuropathic pain (OR = 1.159, 95% CI: 1.112-1.208, p < 0.001). Vitamin B12 deficiency (OR = 0.733, p = 0.611) and low ferritin levels (OR = 1.688, p = 0.349) were not independently significant predictors after adjustment for other variables. These results highlight disease severity, measured by FIQR, as the strongest predictor of neuropathic pain in patients with fibromyalgia, as demonstrated in Table 4.

**Table 4:** Multivariable logistic regression analysis of predictors of neuropathic pain (dependent variable: neuropathic pain = yes) Multivariable logistic regression analysis was performed to identify predictors of neuropathic pain. Chi-square statistics were used to assess the significance of individual predictors. P-value < 0.05 was considered statistically significant. Significant p-values are marked with an asterisk (*). FIQR: Fibromyalgia Impact Questionnaire-Revised

Variable	Coefficient (β)	Odds Ratio (OR)	95% CI for OR	Wald χ^2^*	p-value
Age (per year)	-0.0068	0.993	0.974-1.013	0.45	0.502
Female (vs. male)	0.0971	1.102	0.524-2.318	0.07	0.798
FIQR (per unit)	0.1475	1.159	1.112-1.208	32.84	<0.001*
B12 deficiency (yes vs. no)	-0.3102	0.733	0.222-2.426	0.26	0.611
Low ferritin (yes vs. no)	0.5234	1.688	0.564-5.050	0.88	0.349

## Discussion

Micronutrient deficiencies, particularly serum vitamin B12 and ferritin, have been implicated in altered nerve function and pain modulation [16]. Emerging research suggests that low levels of these biomarkers may influence disease severity and neuropathic manifestations in FMS. However, evidence remains inconsistent across populations, with limited studies from South Asia, particularly Pakistan. Exploring this relationship may help clarify underlying mechanisms and guide adjunctive therapeutic strategies. In our study, neuropathic pain was identified in 58.5% of patients, reinforcing emerging evidence that neuropathic components may be intrinsic rather than incidental to the fibromyalgia phenotype. Micronutrient dysregulation has been increasingly linked to neurosensory dysfunction in chronic pain disorders. Our findings of significantly lower vitamin B12 (275.8 ± 66.8 pg/mL vs. 308.1 ± 69.9 pg/mL; p = 0.001) and ferritin levels (59.1 ± 26.1 ng/mL vs. 68.9 ± 27.2 ng/mL; p = 0.005) in neuropathic patients support the hypothesis that impaired energy metabolism and disrupted myelination may contribute to heightened neural excitability. This mirrors Bingol et al., who demonstrated inverse correlations of B12 (r = -0.190; p = 0.047) and ferritin (r = -0.256; p = 0.007) with FIQ scores [17].

Vitamin B12 deficiency (<200 pg/mL) was detected in 8.7% of our sample, lower than the 42.4% reported by Munipalli et al. using a higher threshold (<400 ng/L) [18]. However, Younas et al. found no significant association between gender and vitamin B12 deficiency in trigeminal neuralgia patients, suggesting that low B12 levels may not be influenced by sex in neuropathic conditions [19]. Consistent with this, Nadeem et al. reported a profound negative association between B12 levels and diabetic neuropathy (coefficient: -115.6; p < 0.001) [20], corroborating our finding that neuropathic manifestations may arise before overt deficiency. Contrary to studies reporting no relationship of B12 and ferritin with neuropathic status [21], our logistic regression showed FIQR score, not biomarkers, as the independent predictor (OR = 1.159; p < 0.001). This implies that micronutrient insufficiency may function as a permissive rather than causative contributor, accelerating neural sensitization only in already severe phenotypes. Our findings converge strongly with Kucuk et al., who identified low vitamin B12 (p = 0.010) and ferritin (p < 0.001) as independent risk factors for fibromyalgia through impaired mitochondrial performance and increased oxidative stress [22]. However, Bakılan et al. found no significant group differences [23]. Complementing this, Baygutalp et al. further highlighted iron-deficiency in 28% of fibromyalgia cases, reinforcing ferritin insufficiency as a clinically relevant concern [24]. Baygutalp et al. reported no statistically significant gender-based difference in serum vitamin B12 or ferritin levels among fibromyalgia patients, indicating that micronutrient status alone may not explain female predominance in symptom severity [24]. These insights reinforce the biological foundation for routine micronutrient screening and personalized correction strategies, as supported by Tarsitano et al. [25,26].

Limitations

This study has certain limitations that should be acknowledged. First, its cross-sectional design limits the ability to establish causal relationships between serum vitamin B12 and ferritin levels and neuropathic pain in patients with fibromyalgia; therefore, temporal associations cannot be determined. Second, as the study was conducted at a single tertiary care center using a non-probability consecutive sampling technique, the findings may have limited generalizability to the broader population. Additionally, certain clinical variables that may influence neuropathic pain and disease severity, such as body mass index (BMI), duration of fibromyalgia symptoms, medication use, and overall nutritional status, were not assessed in the present study and may have acted as potential confounding factors. Due to the cross-sectional design of the study, causal relationships cannot be established, and the observed findings should be interpreted as associations.

## Conclusions

Neuropathic pain is common in fibromyalgia and is strongly linked with greater disease severity. Although vitamin B12 and ferritin deficiencies showed trends toward association, only disease severity emerged as an independent predictor. These findings emphasize the importance of comprehensive evaluation and highlight the need for further longitudinal and interventional studies.
